# Scoliidines: Neuroprotective Peptides in Solitary Scoliid Wasp Venoms

**DOI:** 10.3390/toxins16100446

**Published:** 2024-10-17

**Authors:** Carlos Alberto-Silva, Fernanda Calheta Vieira Portaro, Roberto Tadashi Kodama, Lais Gomes, Brenda Rufino da Silva, Felipe Assumpção da Cunha e Silva, Ken-ichi Nihei, Katsuhiro Konno

**Affiliations:** 1 Natural and Humanities Sciences Center, Experimental Morphophysiology Laboratory, Federal University of ABC (UFABC), São Bernardo do Campo 09606-070, SP, Brazil; brendarufino33@gmail.com (B.R.d.S.); fhellcunha@gmail.com (F.A.d.C.e.S.); 2 Laboratory of Structure and Function of Biomolecules, Butantan Institute, São Paulo 05503-900, SP, Brazil; fernanda.portaro@butantan.gov.br (F.C.V.P.); pararoberval@gmail.com (R.T.K.); lais.gomes@icb.usp.br (L.G.); 3 School of Agriculture, Utsunomiya University, Utsunomiya 321-8505, Tochigi, Japan; nihei98@cc.utsunomiya-u.ac.jp; 4 Institute of Natural Medicine, University of Toyama, Toyama 930-0194, Toyama, Japan

**Keywords:** solitary scoliid wasp, comprehensive analysis, venom, neuroprotective peptides

## Abstract

A comprehensive LC-MS study examined the venom components of the solitary scoliid wasp *Scolia oculata*. Online mass fingerprinting showed that crude venom contains 25 small molecules (amino acids, biogenic amines, and nucleosides/nucleotides) and 45 peptides with MW 400-2700. The small molecules were identified by elemental composition analysis, and peptide sequences were determined by ESI-MS/MS and MALDI-TOF/TOF MS analyses. As major peptide components, a known peptide, β-scoliidine (DYVTVKGFSPLRKA), and three new peptides, γ-scoliidine (YVTVKGFSPLR), δ-scoliidine (YVTVKGFSPLREP) and ε-scoliidine (DYVTVKGFSPLREP) were identified, all of which are closely homologous to each other. Once the neuroprotective effects of β-scoliidine have already been described, the other three new scoliidine peptides were analyzed against oxidative stress-induced toxicity in PC12 neuronal cells by mitochondrial metabolism assay, and the structure-activity relationship was evaluated. Interestingly, pre-treatment with ε-scoliidine increased the mitochondrial metabolism of PC12 cells (106 ± 3.6%; *p* = 0.007) exposed to H_2_O_2_-induced oxidative stress in contrast to γ- and δ-scoliidines (77.6 ± 4.8 and 68.5 ± 4.1%, respectively) in compared to cells treated only H_2_O_2_ (75.8 ± 2.4%). These new peptides were also analyzed for enzyme inhibitor/substrate assays with angiotensin-converting enzyme (ACE), neprilysin (NEP), and acetylcholinesterase (AChE). In these assays, only δ- and ε-scoliidines increased the AChE activity (128.7 ± 3.8%; *p* = 0.01; and 116.8 ± 3.8% *p* = 0.03; respectively) in relation to basal activity (100.1 ± 1.6%). In addition, the four peptides were analyzed through in silico analysis, and none of them demonstrated possible hemolytic and toxic activities. In our study, the comprehensive LC-MS and MS/MS analyses of *Scolia oculate* venom identified four major peptide components of the venom β-, γ-, δ- and ε-scoliidines, and small differences in their primary structures are important to their neuroprotective properties.

## 1. Introduction

Wasps belong to Hymenopteran insects and have venoms to utilize for prey capture and self-defense [1]. They can be divided into two groups according to their lifestyle: social and solitary lifestyle. Hornets and paper wasps belong to the former group, social wasps, having a social lifestyle: living together in large nests. Their venoms are used to defend themselves and their larvae in the nests from predators. These venoms and their components have been well documented, which revealed they are a rich source of bioactive peptides, for example, mastoparans and wasp kinins [2,3,4,5]. Solitary wasps have a solitary lifestyle, living alone separately without having a nest, and the use and function of their venoms are very different from those of social species. Their venoms are utilized for prey (insects and spiders) capture, paralyzing the prey to feed their larvae [1,6]. In contrast to social wasp venoms, solitary wasp venoms have been studied much less recently. That may come from their solitary lifestyle. A large amount of venom is needed for chemical and pharmacological investigation, but it is very difficult to find and collect a sufficient number of wasp individuals since they are living alone separately. In recent years, however, remarkable advances in analytical techniques using mass spectrometry (LC-MS and proteomic analysis) and next-generation DNA sequencing (transcriptomics) made it possible to analyze with a minute amount of venom, which prompted much progress in studies of solitary wasp venoms in last decade [7,8,9,10,11,12,13,14,15].

In order to lead paralysis, solitary wasp venoms may have components associated with neurological systems. Pioneering studies revealed that solitary wasp venoms indeed contain neurotoxins. Philanthotoxins in a digger wasp venom are acylpolyamines that act as non-competitive antagonists of glutamate and nicotinic receptors [16,17], and bradykinin-related peptides blocking acetylcholine (ACh) receptors of insect central nervous system have been found in European scoliid wasp venoms [18,19,20].

With an interest in neuroactive components, we have surveyed a variety of solitary wasp venoms inhabiting Japan for the last few decades. As a result, we have found not only peptide neurotoxins blocking sodium and ASIC channels but also α-helical antimicrobial peptides, bradykinin-related peptides, and FMRFamide-like neuropeptides [21,22,23,24]. Thus, these studies indicate that solitary wasp venoms are a rich source of peptides with a wide range of biological activities.

In recent years, our investigation has focused on scoliid wasp venoms. Scoliid wasps hunt and sting beetle larvae under the ground. Comprehensive LC-MS and MS/MS analysis of the venom extract from *Scolia decorata ventralis* identified two major peptide components named α- and β-scoliidine [25]. Of these, only β-scoliidine showed a neuroprotective effect (protective effects against oxidative stress-induced neurotoxicity in PC12 cells). This is the first case of solitary wasp venom peptides showing neuroprotective activity. Similarly, venom components of *Campsomeriella annulata annulata* were investigated, which led to the identification of two major structural types of peptides, bradykinin related-peptides (α- and β-campsomerin) and linear α-helical peptides (annulatin) [26]. Only α-campsomerin showed a cell growth potentiating effect in neuronal PC12 cells, whereas β-campsomerin had no effect. Annulatin, the first linear α-helical peptides described in scoliid wasp venoms, showed histamine-releasing activity from mast cells.

In this study, we continued the characterization of components of solitary wasp venoms, investigating in detail, for the first time, the composition of *Scolia oculate* venom, a scoliid wasp that inhabits Japan. Comprehensive LC-MS and MS/MS analyses of the crude venom extracts identified four major peptide components, β-, γ-, δ- and ε-scoliidines. These peptides were analyzed against oxidative stress-induced toxicity in PC12 neuronal cells by mitochondrial metabolism assay, and the structure-activity relationship was evaluated, which revealed that small differences in their primary structures are important to their neuroprotective properties.

## 2. Results

### 2.1. Studies of Crude Venom Extract by Liquid Chromatography-Electrospray Ionization-Mass Spectrometry (LC-ESI-MS)

#### 2.1.1. On-Line Mass Fingerprinting

**Figure 1:**
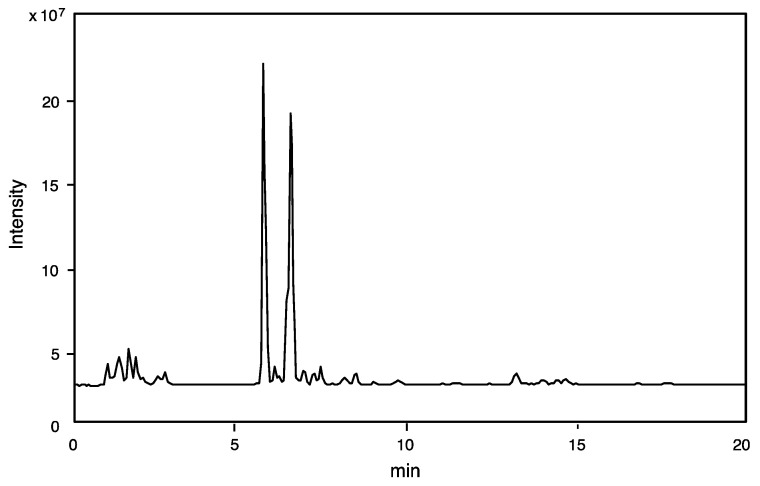
TIC profile was obtained from LC-ESI-MS for the crude venom extract of *Scolia oculata* by reverse-phase HPLC using CAPCELL PAK C_18_ (1.5 × 150 mm) with a linear gradient of 5–65% CH_3_CN/H_2_O/0.1% formic acid over 20 min at flow rate of 200 μL/min.

The crude venom extract was subjected to LC-ESI-MS, and the obtained MS data was analyzed to prepare the component profile (number of components and its molecular mass determination). The total ion current (TIC) profile is shown in Figure 1. Only 50% of the amount of venom sac extract from a single specimen was sufficient for LC-ESI-MS analysis (mass fingerprinting and peptide sequencing).

TIC (total ion chromatogram) was used to create an online mass fingerprint by “virtual fractionation” of MS spectra from a given retention time range (fractions). Each fraction’s molecular mass (M + H)^+^ was computed (Table 1). From 15 virtual fractions with molecular masses from 90 to 2700, 70 components were discovered. The 24 low molecular mass components (*m*/*z* 90–400) may represent free amino acids, biogenic amines, and nucleic acids, while the 45 peptides (*m*/*z* 400–2700) suggest that this venom’s main components are tiny peptides.

#### 2.1.2. Identification of Small Molecules

The identification of small molecules was mostly made by elemental composition analysis of molecular ion (M + H)^+^ with an error range of 0.005 Da. The results are summarized in Table 2, Table 3 and Table 4. In some cases of amino acids, concomitant detection of iminium ion peak supported the identification (Table 2). Similarly, the deamination peak (-NH_3_) strongly supported the structure of biogenic amines (Table 3). For nucleotides (AMP, GMP, IMP, and NAD), analysis of MS/MS spectra obtained by data-dependent MS/MS measurement confirmed their structure (Table 4).

Many of these small molecules are well-known physiologically active compounds; for example, glutamic acid, histamine, and dopamine are neurotransmitters; adenosine is an agonist of corresponding receptors; NAD is a coenzyme. At least some of these small molecules found in this solitary wasp venom may play a role in venom toxic functions, but it remains to be studied.

#### 2.1.3. Peptide Sequencing

MS/MS spectra from 31 peptide molecules were obtained using data-dependent measurement. Manual sequence analysis of these MS/MS spectra yielded the entire sequences of 25 peptides and partial sequences of 6. The studied complete sequences are in Table 5. Table 6 classifies short peptides by structural similarity. They are broadly divided into two classes: bradykinin-related peptides and miscellaneous (no homology nor similarity to any known peptides). Within each class, small differences are found both at N- and C-terminus. It is not sure whether all of them are originally contained in the venom or if some of them are cleavage products from a larger peptide in some way.

As major peptide components, scoliidines (β-, γ, δ, ε-scoliidines) were identified. Of these, β-scoliidine (Fr. 9, RT 5.95, *m*/*z* 1580.873, DYVTVKGFSPLRKA) is already known from our previous work on *Scoliia decorata ventralis* venom showing neuroprotective activity [22]. Other three peptides, γ-scoliidine (Fr. 9, RT 6.49, *m*/*z* 1266.718, YVTVKGFSPLR); δ-scoliidine (Fr. 11, RT 6.84, *m*/*z* 1492.811, YVTVKGFSPLREP); ε-scoliidine (Fr. 13, RT 7.49, *m*/*z* 1607.836, DYVTVKGFSPLREP) are new and closely related to each other. They all have only one L (leucine) residue in their sequence. The MALDI TOF/TOF spectra showed *Wa* ion peak: γ-scoliidine, *m*/*z* 229.1 (*W_a_*_2_); δ-scoliidine *m*/*z* 455.3 (*W_a_*_4_); ε-scoliidine *m*/*z* 455.1 (*W_a_*_4_), which clearly showed that the residues at these positions are L (leucine), not I (isoleucine). These new peptides were synthesized and were identical to the natural peptides (HPLC profile and MS/MS spectra), which corroborated the deduced structure. Since their sequences are comparable to bradykinin-related peptides, these peptides could be associated with them. Seemingly, bradykinin-related peptides are widely distributed in solitary wasp venoms (Table 7). Several minor peptides were identified, for example, GVSKPSWHRDA-NH_2_ (Fr. 2, RT 2.73, *m*/*z* 1238.636), SLGGGVGGLGGLRG-NH_2_ (Fr. 10, RT 6.27, *m*/*z* 1155.655), pQLFTKPSGNEGLRLP (Fr. 15, RT 8.37, *m*/*z* 1639.873). These peptides are distinctive because their sequences are unrelated to known peptides. The biological properties of these novel peptides are remained to be studied.

### 2.2. Biological Characterization of γ-, δ- and ε-Scoliidines

#### 2.2.1. Toxicological Profile in Neuronal PC12 Cells

**Figure 2:**
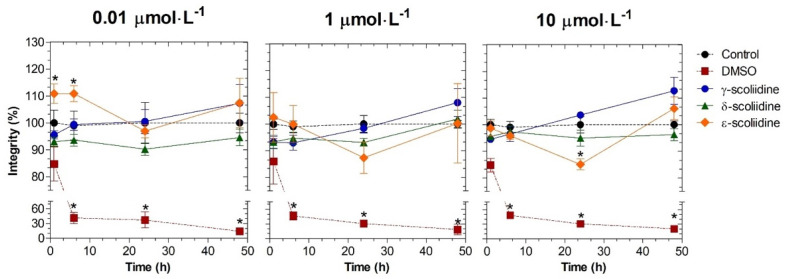
Cytotoxic effects of γ-, δ- and ε-scoliidines on PC12 viability. The cells were exposed to peptides at different concentrations and times. The control and DMSO groups correspond to cells that have not been treated and cells that have been treated with a 5% concentration of DMSO, respectively. Data were collected from three separate experiments in triplicate and demonstrated as the mean ± SEM. Statistical analysis was conducted using a one-way analysis of variance (ANOVA), followed by Dunnett’s post-test. The statistical difference when compared to the control group (*p* < 0.05) was indicated by asterisks.

The peptide ε-scoliidine enhanced cell viability at 0.01 μmoL·L^−1^ for up to 6 h of treatment. However, when the concentration was raised to 10 μmoL·L^−1^ and the treatment extended to 24 h, cell viability decreased (Figure 2). The cell viability remained unchanged at all concentrations and time times assessed when treated with *γ*- and *δ*-scoliidines (Figure 2). The decrease in cell viability was dependent on the concentration of peptide named *ε*-scoliidine, as shown in Figure 2. The addition of DMSO at a concentration of 5% (*v·v^−^*^1^) resulted in a decrease in cell viability in the PC12 cell line.

#### 2.2.2. Scoliidines-Mediated Neuroprotection

**Figure 3:**
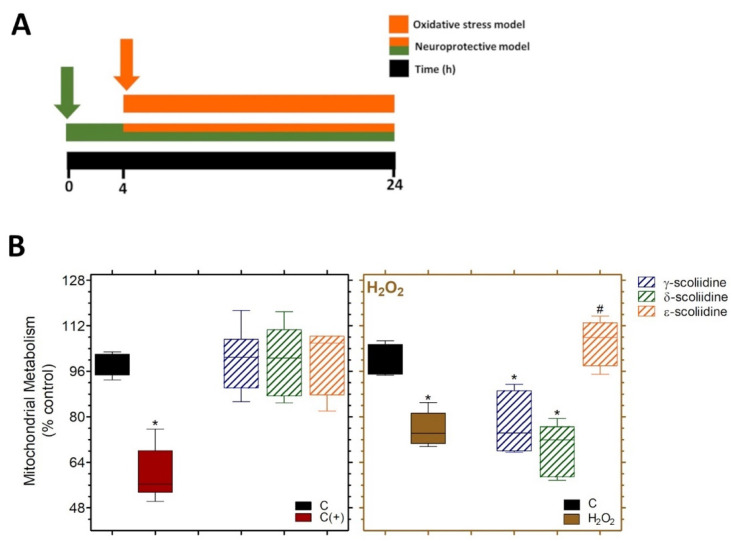
Neuroprotective property of γ-, δ- and ε-scoliidines on mitochondrial metabolism of the PC12 cell line against H_2_O_2_-induced oxidative stress. (**A**) The experiment involved treating cells (5 × 10^3^ cells per well in a 96-well plate) with γ, δ, and ε scoliidines (1 μmol·L^−1^) for 4 h at 37 °C. After that, the medium was changed with a solution containing peptide (1 μmol·L^−1^) and H_2_O_2_ (0.5 mmol·L^−1^), and the cells were incubated for an additional 20 h. (**B**) Protective effects of peptides against neurotoxicity caused by oxidative stress. The data from three separate experiments, each conducted six times, were presented in box-and-whisker plots as percentages relative to the control. A one-way ANOVA was followed by Dunnett’s post-test for statistical analyses. * *p* < 0.05 for differences between the control [C] and experimental groups, and # in relation to the H_2_O_2_ group. C (+) represents cells treated with acrylamide at 100 mmol·L^−1^.

PC12 cells were pre-treated with *γ*-, *δ*- or *ε*-scoliidines for 4 h and then exposed to *γ*-, *δ*- or *ε*-scoliidines containing H_2_O_2_ for an additional 20 h, providing a protective model (Figure 3A). Cells subjected to a 20-h exposure to H_2_O_2_ represent an oxidative stress model. At a concentration of 1 μmoL·L^−1^, all scoliidines did not have any effect on mitochondrial metabolism, compared to acrylamide, which was used as a positive control. Cells treated with H_2_O_2_ (0.5 mmol·L^−1^) showed a significant decrease in mitochondrial metabolism, reducing it to 75.8 ± 2.4% compared to the control group (Figure 3B). *ε*-scoliidines significantly restored the oxidative stress-induced effects, while *γ*- and *δ*-scoliidines did not exhibit neuroprotective effects (Figure 3B).

#### 2.2.3. Effects of γ-, δ- and ε-Scoliidines on AChE Activity

**Figure 4:**
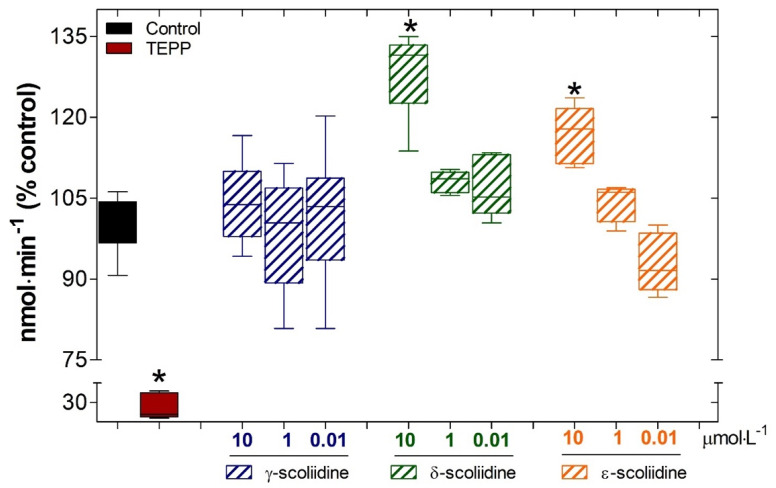
Effects of γ-, δ- and ε-scoliidines on the function of acetylcholinesterase (AChE). The AChE activity was quantified as a percentage of hydrolyzed acetylthiocholine iodine substrate compared to the control (C; blank box). The data obtained from three separate experiments, each performed three times, is presented as mean ± SD. This data was then analyzed using a statistical method, one-way ANOVA, followed by Dunnett’s post-test. * *p* < 0.05 for differences in relation to the control group; TEPP, which corresponds to tetraethyl pyrophosphate, is shown by the red box.

The AChE activity was raised in a concentration-dependent manner by *δ*- and *ε*- scoliidines, as compared to the basal activity. Nevertheless, *γ*- scoliidine did not change the AChE activity (Figure 4). Yet, TEPP was employed as a positive control and effectively decreased the AChE activity to 27.8 ± 6.5% compared to the control (100 ± 1.6%) (Figure 4).

#### 2.2.4. Effects of γ-, δ- and ε-Scoliidines on ACE and NEP Activities

The four synthetic scoliidines were not cleaved by either angiotensin-converting enzyme (ACE-I) or neprilysin (NEP). Furthermore, the peptides were not able to inhibit the catalytic activity of the two metallopeptidases studied (Table 8). Taken together, the results indicate that scoliidines do not interact with the neuropeptidases ACE-I and NEP.

### 2.3. In Silico Analyses

#### 2.3.1. Hemolytic Activity

The results indicate that the peptides do not exhibit hemolytic activity, as shown in Table 9. Indolicidin (ILPWKWPWWPWRR) served as the positive control. The PROB score is the normalized SVM score that ranges from 0 to 1. A value of 1 implies a high chance of being hemolytic, while a score of 0 shows a low possibility.

#### 2.3.2. Toxic Activity

The results presented in Table 10 indicate that the analyzed peptides do not exhibit toxic activity. The positive control utilized in the experiment was Mµ-conotoxin SxIIIA (RCCTGKKGSCSGRACKNLKCCA). The PROB is the SVM score that has been normalized. A score of 1 or greater suggests an increased probability of being injurious, whereas sequences with a value of 0 or less indicate a low chance.

## 3. Discussion

In this study, we investigated in detail the venom components of *Scolia oculata*, which is a continuation of our investigations of the composition of solitary scoliid wasps, the venoms that inhabit Japanese territory. Comprehensive LC-MS and MS/MS analyses of the crude venom extracts identified four major peptide components, β-, γ-, δ- and ε-scoliidines, which can be classified into bradykinin-related peptides due to similarities between their primary structures. In previous studies by our group, we observed the presence of β-scoliidine in the venom of the solitary wasp *Scolia decorata ventralis*, indicating that this peptide may be a common constituent of scoliid wasp venoms [25]. The other three scoliidines reported in the present study, which show great similarities between their primary structures, are unprecedented results.

Scoliid wasp venoms were the first solitary wasp venoms to be studied between the years 1987 and 1990, and the presence of bradykinin-related peptides was reported. The peptides called Megascoliakinin and Thr^6^-bradykinin (Thr^6^-BK) were isolated from the venoms of the European scoliid wasps *Megascolia flavifrons* and *Colpa interrupta* (Table 7), showing a blocking action on ACh receptors in the insects’ central nervous system ACh receptors of insect central nervous system [18,19,20]. These pioneering works proved that solitary wasps have neuroactive components in their venoms. Since the functional role of solitary wasp venoms is fundamentally to paralyze their prey (insects and spiders), it is believed that the venoms contain neuroactive compounds. Therefore, this is the main focus of the present study.

In this scenario, we have surveyed a variety of solitary wasp venoms inhabiting Japan for the last few decades, which resulted in finding not only peptide neurotoxins blocking sodium and ASIC channels but also α-helical antimicrobial peptides, bradykinin-related peptides and FMRFamide-like neuropeptides [21,22,23,24]. The experiments were done using traditional methods, which included HPLC purification followed by chemical analysis of the obtained products. Despite the presence of a vast number of components in venoms, only a limited number of significant components were effectively isolated and described using this method. Furthermore, a major limitation of these studies is the significant amount of venom extracts required. Thus, conducting these studies needs a collection of a number of individuals, which is generally difficult due to the lifestyle of solitary wasps and, in particular, in the case of scoliid wasps, as they hunt and sting beetle larvae under the ground.

Remarkable advances in analytical techniques using mass spectrometry (LC-MS and proteomic analysis) and next-generation DNA sequencing (transcriptomics) made it possible to analyze with a minute amount of venom, which prompted much progress in studies of solitary wasp venoms in the last decade [7,8,9,10,11,12,13,14,15]. We reported venom component analysis of solitary scoliid wasps *Scolia ventralis* and *Campsomeriella annulata annulata* using LC-MS [25,26]. This highly efficient analytical means was used again for this study. Comprehensive analysis of the *Scolia oculata* venom extract by using LC-ESI-MS achieved the profile of its components, consisting of 70 molecules. A majority of them are small peptides, and manual analysis of their MS/MS spectra led to the determination of the full sequence of 25 peptides. They are broadly classified into two major structural types: bradykinin-related peptides and uncharted sequences.

Three types of scoliid wasp venoms have been examined, and all of them include bradykinin-related peptides. These are frequently found in the venoms of scoliid wasp venoms. Based on this thorough examination, it was possible to identify small molecules such as amino acids, biogenic amines, and nucleosides/nucleotides, which had been previously described in studies [25,26]. Some of them have been reported as functional components present in solitary wasp venoms, such as histamine contributing to pain-producing activity [28]. The venom of the emerald jewel wasp *Ampulex compressa* contains dopamine, which has a role in the distinctive behavior of its victim, the American cockroach [10]. However, the specific physiological roles of this venom have not yet been determined. Similarly, it is possible that any other small molecules discovered in the venom of *Scolia oculata* may elicit physiological responses when introduced into the larval prey of the beetle. However, further investigation is required to confirm this hypothesis. It is important to mention that peptides obtained from venoms have been used as the basis for the development of potential therapeutic candidates and innovative therapies [29,30]. Recent research suggests that peptides obtained from natural sources or their synthetic equivalents, such as wasp venoms, have demonstrated potential options for neuroprotection against oxidative stress [25,26]. The β-scoliidine-mediated neuroprotection was first reported in our previous work with the *Scoliia decorata ventralis* venom [25]. It is noteworthy that the venoms of different solitary wasps have this identical molecule in their venoms, possibly indicating an important biological function for these insects.

Remarkably, prior administration of ε-scoliidine enhanced the mitochondrial metabolism of PC12 cells when subjected to oxidative stress generated by H_2_O_2_, in contrast to γ- and δ-scoliidines as compared to cells treated just with H_2_O_2_. Also, our findings indicate that only ε-scoliidine significantly restored the oxidative stress-induced neurotoxicity in contrast to γ- and δ-scoliidines, despite their substantial similarity. It is possible that residues D and Y present at the N-terminus of ε-scoliidine is responsible for this activity. It is worth noting that the neuroprotective activities of scoliidines were obtained under conditions of oxidative stress induced by H_2_O_2,_ and different experimental models should be used to verify the extent of neuroprotection induced by this class of neuropeptides.

Thinking about the potential use of β- and ε-scoliidines as prototypes of molecules with neuroprotective activities, new experiments should be performed with various concentrations to better evaluate the attained results since a single dose of each of the scoliidines was used and may not reflect the real physiological condition.

In silico, analyses were carried out in relation to their possible hemolytic and toxic actions. In fact, none of the scoliidines showed potential for such activities, but in varying degrees. Once again, we observe that small differences in the primary structures in this family of peptides result in different activities. It is important to emphasize that the results obtained with the in silico analyses are preliminary, and further experiments should be carried out to confirm the lack of toxic and hemolytic activities of the peptides under study.

Peptides are attracting significant interest as physiologically significant compounds, particularly as modulators of acetylcholinesterase (AChE), and therefore, the scoliidines reported here were studied as possible modulators of the activity of this enzyme. AChE (E.C.3.1.1.7) facilitates the breakdown of the neurotransmitter ACh into choline and acetic acid [31]. Inhibiting AChE would result in elevated amounts of ACh in the brain, leading to enhanced cholinergic connections in individuals with Alzheimer’s disease [32,33]. To our surprise, δ- and ε-scoliidines resulted in the activation of the enzyme’s catalytic activity but not γ-scoliidine. It is possible that this observed activity is important for the predation of larvae by the *Scolia oculata* wasp. However, further studies will be necessary to understand this enzymatic activation.

Since scoliidines show similarity between their primary sequences and bradykinin (RPPGFSPFR), the possible interactions of the synthetic peptides with the metallopeptidases ACE-I and NEP were investigated. Furthermore, ACE-like and NEP-like enzymes have already been described as important neuropeptidases present in insects, including wasps [34]. As previously reported for β-scoliidine [25], γ-scoliidine, δ-scoliidine, and ε-scoliidine also did not behave as substrates or inhibitors of these peptidases.

## 4. Conclusions

Four major peptide components, α-, β-, γ, and δ-scoliidines, were identified in the venom extracts from the solitary scoliid wasp *Scolia oculata* by comprehensive LC-MS and MS/MS analysis. The peptides were evaluated for their impact on oxidative stress-induced toxicity in PC12 neuronal cells using a mitochondrial metabolism evaluation, and the structure-activity relationship was evaluated, which revealed that small differences in their primary structures are important to their neuroprotective properties.

## 5. Materials and Methods

### 5.1. Wasp Collection

In August 1996, a total of nine female specimens of *Scolia oculata* were hand-collected in Kanagawa, Japan, using an insect-catching net. The venom sacs were dissected while the subject was under low-temperature anesthesia and then extracted using a solution consisting of 50% MeCN (acetonitrile) and water. The extracts were freeze-dried and kept at −35 °C until they were ready to be used. Just prior to use, the lyophilized extracts were dissolved into 180 μL of water at room temperature, and immediately after preparation, 10 μL of this solution, corresponding to 50% of the amount of venom sac extracts from a single specimen, was injected into HPLC.

### 5.2. Reagents and Cell Line

All chemicals used here were analytical reagent grade (purity higher than 95%) purchased from different brands. The AChE enzyme was derived from *Electrophorus electricus* Type VI-S, and the ACE I enzyme was derived from rabbit lung and was acquired from Sigma-Aldrich Corporation (St. Louis, MO, USA). Neprilysin and the substrates [Abz-FRK (Dnp) P-OH and Abz-RGFK (Dnp)-OH] were supplied by Prof. Adriana Carmona from the Department of Biophysics at Federal University of São Paulo (SP, Brazil). PC12 cell (American Type Culture Collection ^®^ CRL-1721™; Manassas, VA, USA) was used in the present study. Cells were routinely cultured in DMEM medium (Sigma-Aldrich Corporation; St. Louis, MO, USA), according to a previous study [26].

### 5.3. LC-ESI-MS

The crude venom was evaluated using an LC (Accela 600 Pump, Thermo Fisher Scientific, Waltham, MA, USA) and ESI-FTMS (LTQ Orbitrap XL, Thermo Fisher Scientific, Waltham, MA, USA) systems, according to reports in the literature [25,26]. The lyophilized venom was subjected to reversed-phase HPLC using CAPCELL PAK C_18_ (SHISEIDO Co., Ltd., Tokyo, Japan). ESI-FTMS was operated by Xcalibur^TM^ software, Version 2.5 (Rev. 2) (Thermo Fisher Scientific, Waltham, MA, USA), and MS/MS spectra were obtained by data-dependent MS/MS mode) and the obtained spectra were manually analyzed to give peptide sequences. They were confirmed by MS-Product in the ProteinProspector program (https://prospector.ucsf.edu/prospector/mshome.htm) accessed on 5 October 2022.

### 5.4. MALDI-TOF MS

Autoflex TOF/TOF mass spectrometers (Bruker Daltonics, Yokohama, Japan) with 335 nm pulsed YAG laser reflector mode were used to collect MALDI-TOF MS spectra. All spectra were acquired according to the procedure reported by the previous study [25,26]. Mix 0.5 µL of sample solution on the MALDI plate with 0.5 µL of matrix solution and let dry at room temperature. The collision gas for TOF/TOF measurement was argon, and ions were accelerated at 19 kV. The series of *b* and *y* ions allowed manual amino acid sequence identification.

### 5.5. Peptide Synthesis

GenScript (Nanjing, Jiangsu, China) produced the peptides using Fmoc chemistry. RP-HPLC, using a preparative C18 column, purified the crude products, and HPLC and MS confirmed the resulting peptides’ purity and molecular weight.

### 5.6. ACE and NEP Activities

Different concentrations of peptides (20, 30, and 50 μmol·L^−1^) containing ACE, NEP, and their FRET substrates (2, 4, and 8 μmol·L^−1^), FRK(Dnp)P-OH and Abz-RGFK (Dnp)-OH, were tested, according to a previous study [25,26]. To conduct the experiments, both peptidases and substrates were added to an appropriate buffer and incubated at 37 °C. All experiments included peptide-free controls. Fluorimeter (Victor 3-Perkin–Elmer) monitored reactions for 15 min, and Erithacus Software’s GraFit 3.0 evaluated the findings. The experiments were performed in triplicate.

### 5.7. Stability Tests of Peptides

The stability tests of peptides (30 µmol·L^−^^1^) were tested after incubation separately with ACE and NEP (7.5 ng) at 37 °C for 4 h, according to previous reports [25,26].

### 5.8. Measurement of AChE Activity

The inhibition of AChE activity was determined spectrophotometrically based on Ellman’s method, as previously reported [35]. We calculated AChE activity as the hydrolyzed acetylthiocholine iodide substrate concentration as a percentage of control.

### 5.9. Toxicity Studies on the Integrity Cell

The cytotoxicity assay was performed using the staining of attached cells with crystal violet dye, according to the literature [26,36]. Briefly, PC12 cells (5 × 10^3^ cells per well; 96-well plates) were treated with different concentrations and times. Untreated cells or treated with DMSO (5%; *v*·*v*^−1^) were included as controls. We measured the absorbance of dye solubilized in methanol at 570 nm using a microplate reader (BioTek Instruments, Inc., Winooski, VT, USA). We collected data from three distinct examinations, each performed three times, showing the percentage of cell viability relative to the control.

### 5.10. Effects of Scoliidines on Changes Induced by Oxidative Stress

PC12 cells (5 × 10^3^ cells per well) seeded in a 96-well plate (Nest Biotechnology, Rahway, NJ, USA) were used to study the neuroprotective effects of peptides against H_2_O_2_-induced oxidative stress, according to a previous study [25,26]. In summary, cells were treated with peptides at 1 µmol·L^−1^ for 4 h. The mediums were replaced by medium containing the peptides and H_2_O_2_ (0.5 mmol·L^−1^) and incubated for 20 h more. Cells untreated or treated with H_2_O_2_ were used as controls. The mitochondrial metabolism was analyzed by 3-(4,5-dimethylthiazol-2-yl)-2,5-diphenyltetrazolium bromide (MTT; 0.5 mg·mL^−1^ for 3 h of incubation at 37 °C) reduction assay [37]. The absorbance of the MTT formazan dissolved in 100% DMSO was measured at a wavelength of 540 nm using a spectrophotometer (BioTek Instruments, Inc., Winooski, VT, USA). We represented the data using box-and-whisker plots to show the proportion of mitochondrial metabolism in comparison to the control.

### 5.11. Prediction of Hemolytic Peptides

Synthetic peptide hemolysis was predicted using the HemoPI platform (https://webs.iiitd.edu.in/raghava/hemopi/, accessed on 2 July 2024). The positive control was indolicidin (ILPWKWPWWPWRR), which has wide hemolytic activity. The “SVM (HemoPI-1) based” prediction approach was employed for analysis, and the PROB score ranged from 0 to 1 (where 1 indicates a high chance of hemolysis and 0 indicates a low risk).

### 5.12. Prediction of Toxic Peptides

The ToxinPred platform: ToxinPred Designing and prediction of toxic peptides (http://crdd.osdd.net/raghava/toxinpred/protein.php, accessed on 2 July 2024) was used to predict the toxic activity. The toxic peptide Mµ-conotoxin SxIIIA (RCCTGKKGSCSGRACKNLKCCA) was used as a positive control. The “SVM (Swiss-Prot) based” prediction method was used for the analysis, and the PROB score is the normalized SVM score. Sequences with a score of 1 or higher indicate a high probability of being toxic, while sequences with a score of 0 or lower indicate a low probability.

### 5.13. Statistical Analyses

All results are shown as mean ± SD from three separate experiments (*n* = 3) in triplicate. One-way analysis of variance (ANOVA) was used for between-group comparisons, and Dunnett’s post-hoc test was used for multiple comparisons. Statistical significance was defined as *p* < 0.05. The analyses were done using GraphPad Prism 6.0 (GraphPad Software, Inc., La Jolla, CA, USA).

## Figures and Tables

**Table 1:** Online mass fingerprint by LC-ESI-MS.

Fr. No.	Retention Time (min)	(M + H)^+^ *m*/*z*
1	0.9–1.5	90.053, 106.050, 112.087, 116.070, 118.086, 120.065, 133.061, 134.045, 148.069, 154.086, 156.076, 175.119, 258.110
2	1.5–2.0	132.102, 150.058, 182.081, 268.104, 284.099, 348.069, 349.054, 364.065, 664.109
3	2.0–2.3	269.088, 1270.626
4	2.3–3.0	166.086, 609.359, 666.381, 1053.557, 1082.546, 1238.636
5	3.0–4.0	1237.782, 2693.467
6	4.0–5.0	205.097, 604.344, 962.551, 1091.567, 1306.624
7	5.0–5.4	806.403, 1004.587, 1393.829
8	5.4–5.8	676.377, 1003.596, 1203.717, 1302.786, 1465.848
9	5.8–6.2	1580.874, 1682.892, 2006.099
10	6.2–6.6	813.448, 1004.585, 1103.652, 1155.655, 1266.718, 1545.804
11	6.6–7.0	997.532, 1230.679, 1337.754, 1395.759,1492.811, 2033.062
12	7.0–7.4	1034.511, 1346.671, 1368.653
13	7.4–7.9	1095.686, 1110.615, 1139.648, 1572.767, 1607.836
14	7.9–8.2	—
15	8.2–9.0	1282.551

**Table 2:** Amino acids identified by LC-ESI-MS.

RT (min)	Intensity × 10^4^	[M + H]^+^ *m*/*z*	Elemental Composition	Iminium Ion *m*/*z*	Elemental Composition	Compound
1.07	10	156.076	C_6_H_10_N_3_O_2_	—		Histidine
	35	175.119	C_6_H_15_N_4_O_2_	—		Arginine
1.28	2	90.055	C_3_H_7_NO_2_	—		Alanine
	4	106.050	C_3_H_8_NO_3_	—		Serine
	180	116.070	C_5_H_10_NO_2_	70.065	C_4_H_8_N	Proline
	20	118.086	C_5_H_12_NO_2_	72.081	C_4_H_10_N	Valine
	6	120.065	C_4_H_10_NO_3_	—		Threonine
	4	133.061	C_4_H_9_N_2_O_3_	—		Asparagine
	9	134.045	C_4_H_8_NO_4_	—		Aspartic acid
	100	148.060	C_5_H_10_NO_4_	102.050	C_4_H_8_NO_2_	Glutamic acid
1.55	3	150.058	C_5_H_12_NO_2_S	—		Methionine
1.69	30	132.102	C_6_H_14_NO_2_	86.096	C_5_H_12_N	L/I *
1.76	14	182.081	C_9_H_12_NO_3_	—		Tyrosine
2.56	4	166.086	C_9_H_12_NO_2_	120.081	C_8_H_10_N	Phenylalanine
4.33	1	205.097	C_11_H_13_N_2_O_2_	—		Tryptophan

* Either leucine (L) and/or isoleucine (I).

**Table 3:** Biogenic amines identified by LC-ESI-MS.

RT (min)	Intensity × 10^4^	[M + H]^+^ *m*/*z*	Elemental Composition	Deammonia *m*/*z*	Elemental Composition	Compound
1.07	12	112.087	C_5_H_10_N_3_	95.060	C_5_H_7_N_2_	Histamine
1.34	42	154.086	C_8_H_12_NO_2_	137.059	C_8_H_9_O_2_	Dopamine

**Table 4:** Nucleosides/nucleotides identified by LC-ESI-MS.

RT (min)	Intensity × 10^4^	[M + H]^+^ *m*/*z*	Elemental Composition	Compound
1.28	30	258.110	C_10_H_16_N_3_O_5_	Thymidine
1.63	52	348.069	C_10_H_15_N_5_O_7_P	AMP (Adenosine monophosphate) *
	2	664.109	C_21_H_28_N_7_O_14_P_2_	NAD (Nicotinamide adenine dinucleotide) *
1.69	150	268.104	C_10_H_14_N_5_O_4_	Adenosine
1.76	5	364.065	C_10_H_15_N_5_O_8_P	GMP (Guanosine monophosphate) *
1.98	9	284.099	C_10_H_14_N_5_O_5_	Guanosine
	20	349.054	C_10_H_14_N_4_O_8_P	IMP (Inosine monophosphate) *
2.05	14	269.088	C_10_H_13_N_4_O_5_	Inosine

* Identified by MS/MS analysis.

**Table 5:** Peptide sequences identified by MS/MS spectral analysis.

Fr	RT	Intensity × 10^3^	Precursor Ion *m*/*z* (Charge)	Molecular Mass (M + H)^+^	Sequence
4	2.51	210	305.183 (2+)	609.359	YVTVK
	2.68	77	361.521 (3+)	1082.546	SKPSWHRDA-NH_2_
	2.73	360	413.550 (3+)	1238.636	GVSKPSWHRDA-NH_2_
	2.88	51	333.695 (2+)	666.381	YVTVKG
	2.95	23	351.858 (3+)	1053.557	GVSKPSWHR
7	4.49	36	302.676 (2+)	604.344	FNPKV
8	5.45	18	338.692 (2+)	676.377	GFSPLR
	5.61	183	434.934 (3+)	1302.786	VTVKGFSPLRKA
	5.65	7400	489.288 (3+)	1465.848	YVTVKGFSPLRKA
	5.66	660	401.911 (3+)	1203.717	TVKGFSPLRKA
9	5.95	163	527.629 (3+)	1580.873	DYVTVKGFSPLRKA
	6.06	36	561.636 (3+)	1682.892	pQLFTKPSGNEGLRPR
10	6.27	300	578.332 (2+)	1155.655	SLGGGVGGLGGLGR-NH_2_
	6.34	40	407.228 (2+)	813.448	YVTVKGF
	6.49	13,600	422.911 (3+)	1266.718	YVTVKGFSPLR
11	6.63	150	499.270 (2+)	997.532	YVTVKGFSP
	6.66	18	465.925 (3+)	1395.759	YVTVKGFSPLRE
	6.77	28	446.590 (3+)	1337.754	AYVTVKGFSPLR
	6.84	660	498.276 (3+)	1492.811	YVTVKGFSPLREP
13	7.49	136	536.617 (3+)	1607.836	DYVTVKGFSPLREP
	7.66	25	548.347 (2+)	1095.686	PKLLQSLNAL-NH_2_
	7.81	18	555.812 (2+)	1110.615	YVTVKGFSPLR
15	8.37	790	820.441 (2+)	1639.873	pQLFTKPSGNEGLRLP
	8.39	112	630.789 (3+)	1260.569	pQDDLSDFNPKV
16	9.37	23	629.814 (2+)	1258.620	pQDVDHVFLRF

**Table 6:** Classification of the peptides.

RT	Intensity × 10^3^	(M + H)^+^	Sequence
**Scoliidines (Bradykinin-related peptides)**			
2.51	210	609.359	YVTVK
2.88	51	666.381	YVTVKG
6.34	40	813.448	YVTVKGF
6.63	150	997.532	YVTVKGFSP
7.81	18	1110.615	YVTVKGFSPL
5.45	18	676.377	GFSPLR
6.49	13,600	1266.718	YVTVKGFSPLR (γ-scoliidine)
6.77	28	1337.754	AYVTVKGFSPLR
5.66	660	1203.717	TVKGFSPLRKA
5.61	183	1302.786	VTVKGFSPLRKA
5.65	7400	1465.848	YVTVKGFSPLRKA
5.95	163	1580.873	DYVTVKGFSPLRKA (β-scoliidine)
6.66	18	1395.759	YVTVKGFSPLRE
6.84	660	1492.811	YVTVKGFSPLREP (δ-scoliidine)
7.49	136	1607.836	DYVTVKGFSPLREP (ε-scoliidine)
**Miscellaneous**			
2.95	23	1053.557	GVSKPSWHR
2.73	360	1238.636	GVSKPSWHRDA-NH_2_
2.68	77	1082.546	SKPSWHRDA-NH_2_
8.37	790	1639.873	pQLFTKPSGNEGLRLP
6.06	36	1682.892	pQLFTKPSGNEGLRPR
7.66	25	1095.686	PKLLQSLNAL-NH_2_
6.27	300	1155.655	SLGGGVGGLGGLRG-NH_2_

**Table 7:** Bradykinin-related peptides.

Peptide	Sequence	References
Bradykinin (BK)	RPPGFSPFR	[8,27]
Thr^6^-Bradykinin (Thr^6^-BK)	RPPGFTPFR	[18,19,27]
Megascoliakinin	RPPGFTPFRKA	[20]
α-Campsomerin	PRLRRLTGLSPLR	[26]
β-Campsomerin	PRLRRLTGLSPLRAP	[26]
α-Scoliidine	DYVTVKGFSPLR	[25]
β-Scoliidine	DYVTVKGFSPLRKA	[25] This work
γ-Scoliidine	YVTVKGFSPLR	This work
δ-Scoliidine	YVTVKGFSPLREP	This work
ε-Scoliidine	DYVTVKGFSPLREP	This work

**Table 8:** Evaluation of inhibition and relative hydrolysis ratios for bradykinin and β-, γ-, δ- and ε-scoliidines for angiotensin-converting enzyme (ACE) and human neprilysin (NEP).

	Inhibition (%)		Cleavage (%)	
Peptide	NEP	ACE	NEP	ACE
bradykinin	-	-	100	100
β-scoliidine	<0.01	<0.01	<0.01	<0.01
γ-scoliidine	<0.01	<0.01	<0.01	<0.01
δ-scoliidine	<0.01	<0.01	<0.01	<0.01
ε-scoliidine	<0.01	<0.01	<0.01	<0.01

**Table 9:** Prediction of hemolytic peptides.

Peptides	ProbScore	Prediction
β-scoliidine	0.26	Non-hemolytic
γ-scoliidine	0.37	Non-hemolytic
δ-scoliidine	0.03	Non-hemolytic
ε-scoliidine	0.00	Non-hemolytic
Indolicidin	0.94	Hemolytic

**Table 10:** Prediction of peptide toxicity.

Peptides	ProbScore	Prediction
β-scoliidine	−0.40	Non-toxic
γ-scoliidine	−0.15	Non-toxic
δ-scoliidine	−0.25	Non-toxic
ε-scoliidine	−0.25	Non-toxic
Mµ-conotoxin SxIIIA	2.64	Toxic

## Data Availability

All data generated or analyzed during this study are included in this published article.

## References

[1] Piek T. (1986). Venoms of the Hymenoptera: Biochemical, Pharmacological and Behavioural Aspects.

[2] Nakajima T., Piek T. (1986). Pharmacological Biochemistry of Vespid Venoms. Venoms of the Hymenoptera: Biochemical, Pharmacological and Behavioural Aspects.

[3] Silva J., Monge-Fuentes V., Gomes F., Lopes K., dos Anjos L., Campos G., Arena C., Biolochi A., Gonçalves J., Galante P. (2015). Pharmacological alternatives for the treatment of neurodegenerative disorders: Wasp and bee venoms and their components as new neuroactive tools. Toxins.

[4] Abd El-Wahed A., Yosri N., Sakr H.H., Du M., Algethami A.F.M., Zhao C., Abdelazeem A.H., Tahir H.E., Masry S.H.D., Abdel-Daim M.M. (2021). Wasp venom biochemical components and their potential in biological applications and nanotechnological interventions. Toxins.

[5] Luo L., Kamau P.M., Lai R. (2022). Bioactive peptides and proteins from wasp venoms. Biomolecules.

[6] O’Neill K.M. (2001). Solitary Wasps: Behavior and Natural History.

[7] Lee S.H., Baek J.H., Yoon K.A. (2016). Differential properties of venom peptides and proteins in solitary vs. social hunting wasps. Toxins.

[8] White S.R., Kadavakollu S. (2016). Bradykinin in *Hemipepsis ustulata*: A novel method for safely milking wasps. Toxicon.

[9] Nolasco M., Biondi I., Pimenta D.C., Branco A. (2018). Extraction and preliminary chemical characterization of the venom of the spider wasp *Pepsis decorata* (Hymenoptera: Pompilidae). Toxicon.

[10] Moore E.L., Arvidson R., Banks C., Urenda J.L., Duong E., Mohammed H., Adams M.E. (2018). Ampulexins: A new family of peptides in venom of the emerald jewel wasp, *Ampulex Compressa*. Biochemistry.

[11] Kotea S., Faktorb J., Dapica I., Mayordomoa M.Y., Kocikowskia M., Kagansky A., Goodletta D., Vojtesek B., Huppa T., Wilcockson D. (2019). Analysis of venom sac constituents from the solitary, aculeate wasp *Cerceris rybyensis*. Toxicon.

[12] Huicab-Uribe M.A., Verdel-Aranda K., Martínez-Hernández A., Zamudio F.Z., Jiménez-Vargas J.M., Lara-Reyna J. (2019). Molecular composition of the paralyzing venom of three solitary wasps (Hymenoptera: Pompilidae) collected in southeast Mexico. Toxicon.

[13] Jensen T., Walker A.A., Nguyen S.H., Jin A.-H., Deuis J.R., Vetter I., King G.F., Schmidt J.O., Robinson S.D. (2021). Venom chemistry underlying the painful stings of velvet ants (Hymenoptera: Mutillidae). Cell. Mol. Life Sci..

[14] Dashevsky D., Rodriguez J. (2021). A short review of the venoms and toxins of spider wasps (Hymenoptera: Pompilidae). Toxins.

[15] Nolasco M., Mariano D.O.C., Pimenta D.C., Biondi I., Branco A. (2023). Proteomic analysis of venom from a Spider Hawk, *Pepsis decorata*. J. Venom. Anim. Toxins Incl. Trop. Dis..

[16] Eldefrawi A.T., Eldefrawi M.E., Konno K., Mansour N.A., Nakanishi K., Oltz E., Usherwood P.N.R. (1988). Structure and synthesis of a potent glutamate receptor antagonist in wasp venom. Proc. Natl. Acad. Sci. USA.

[17] Piek T., Hue B. (1989). Philanthotoxins, a new class of neuroactive polyamines, block nicotinic transmission in the insect CNS. Comp. Biochem. Physiol..

[18] Yasuhara T., Mantel P., Nakajima T., Piek T. (1987). Two kinins isolated from an extract of the venom reservoirs of the solitary wasp *Megascolia flavifrons*. Toxicon.

[19] Piek T., Hue B., Mantel P., Nakajima T., Pelhate M., Yasuhara T. (1990). Threonine6-bradykinin in the venom of the wasp *Colpa interrupta* (F.) presynaptically blocks nicotinic synaptic transmission in the insect CNS. Comp. Biochem. Physiol..

[20] Piek T., Hue B., Mony L., Nakajima T., Pelhate M., Yasuhara T. (1987). Block of synaptic transmission in insect CNS by toxins from the venom of the WASP *Megascolia flavifrons* (FAB.). Comp. Biochem. Physiol..

[21] Konno K., Kazuma K., Nihei K. (2016). Peptide toxins in solitary wasp venoms. Toxins.

[22] Cabrera M.P.D.S., Rangel M., Ruggiero Neto J., Konno K. (2019). Chemical and biological characteristics of antimicrobial-helical peptides found in solitary wasp venoms and their interaction with model membranes. Toxins.

[23] Hernández C., Konno K., Salceda E., Vega R., Zaharenko A.J., Soto E. (2019). Sa12b peptide from solitary wasp inhibits ASIC currents in rat dorsal root ganglion neurons. Toxins.

[24] Nihei K., Peigneur S., Tytgat J., Lange A.B., Konno K. (2021). Isolation and characterization of FMRFamide-like peptides in the venoms of solitary sphecid wasps. Peptides.

[25] Alberto-Silva C., Portaro F., Kodama R., Pantaleão H., Rangel M., Nihei K., Konno K. (2021). Novel neuroprotective peptides in the venom of the solitary scoliid wasp *Scolia decorata ventralis*. J. Venom. Anim. Toxins Incl. Trop. Dis..

[26] Alberto-Silva C., Portaro F.C.V., Kodama R.T., Pantaleão H.Q., Inagaki H., Nihei K., Konno K. (2021). Comprehensive analysis and biological characterization of venom components from solitary scoliid wasp *Campsomeriella annulata annulata*. Toxins.

[27] Konno K., Palma M.S., Hitara I.Y., Juliano M.A., Juliano L., Yasuhara T. (2002). Identification of bradykinins in solitary wasp venoms. Toxicon.

[28] Nakajima T., Yasuhara T., Yoshida N., Takemoto Y., Shinonaga S., Kano R., Yoshida H. (1983). The pattern analysis of biologically active amines in some Hymenopteran venoms by high performance liquid chromatography. Med. Entomol. Zool..

[29] de Souza J.M., Goncalves B.D.C., Gomez M.V., Vieira L.B., Ribeiro1 F.M. (2018). Animal toxins as therapeutic tools to treat neurodegenerative diseases. Front. Pharmacol..

[30] Oliveira A.L., Viegas M.F., da Silva S.L., Soares A.M., Ramos M.J., Fernandes P.A. (2022). The chemistry of snake venom and its medicinal potential. Nat. Rev. Chem..

[31] Walczak-Nowicka Ł.J., Herbet M. (2021). Acetylcholinesterase inhibitors in the treatment of neurodegenerative diseases and the role of acetylcholinesterase in their pathogenesis. Int. J. Mol. Sci..

[32] Prasasty V., Radifar M., Istyastono E. (2018). Natural peptides in drug discovery targeting acetylcholinesterase. Molecules.

[33] Teixeira J., de Castro A., Soares F., da Cunha E., Ramalho T. (2019). Future therapeutic perspectives into the Alzheimer’s disease targeting the oxidative stress hypothesis. Molecules.

[34] Isaac R.E., Bland N.D., Alan D., Shirras A.D. (2009). Neuropeptidases and the metabolic inactivation of insect neuropeptides. Gen. Comp. Endocrinol..

[35] Malheiros F.B.M., Vicente M.E., Morales A.G., Alberto-Silva C. (2022). Efficiency of the removal of tetraethyl pyrophosphate (TEPP) pesticide in water: Use of cork granules as a natural adsorbent on acetylcholinesterase activity in neuronal PC12 cell. J. Environ. Sci. Health B.

[36] Feoktistova M., Geserick P., Leverkus M. (2016). Crystal violet assay for determining viability of cultured cells. Cold Spring Harb. Protoc..

[37] Stockert J.C., Blázquez-Castro A., Cañete M., Horobin R.W., Villanueva Á. (2012). MTT assay for cell viability: Intracellular localization of the formazan product is in lipid droplets. Acta Histochem..

